# Evidence of seasonal changes in airborne particulate matter concentration and occupation-specific variations in pulmonary function and haematological parameters among some workers in Enugu Southeast Nigeria: a randomized cross-sectional observational study

**DOI:** 10.1186/s13690-022-00967-3

**Published:** 2022-09-22

**Authors:** Sam Chidi Ibeneme, Rita Nkechi Ativie, Georgian Chiaka Ibeneme, Hellen Myezwa, Amarachi Destiny Ezuma, Amaka Nnamani, Salome Ezeofor, Maduabuchukwu Joseph Nwankwo, Theresa Ucheoma Ettu, Akachukwu Omumuagwula Nwosu, Ifeoma Joy Okoye, Gerhard Fortwengel

**Affiliations:** 1 Department of Medical Rehabilitation, Faculty of Health Sciences, College of Medicine, University of Nigeria, Enugu Campus, Enugu, Enugu State Nigeria; 2 Department of Nursing Sciences, Faculty of Health Sciences & Technology, College of Health Sciences, Ebonyi State University, Abakaliki, Ebonyi State Nigeria; 3 Department of Physiotherapy, Faculty of Health Sciences, School of Therapeutic Studies, University of the Witwatersrand, 7 York Road, Parktown, Johannesburg, Gauteng 2193 South Africa; 4 Department of Physiotherapy, University of Nigeria Teaching Hospital, Ituku/Ozalla, Enugu, Enugu State Nigeria; 5 Department of Radiation Medicine, Faculty of Medical Sciences, College of Medicine, University of Nigeria, Enugu Campus, Enugu, Enugu 400001 Nigeria; 6 Department of Medical Rehabilitation, Faculty of Health Sciences, College of Health Sciences, Nnamdi Azikiwe University, nnewi Campus, Nnewi, Anambra State Nigeria; 7 Owerri Study Centre, National Open University of Nigeria, Owerri, Imo State Nigeria; 8 Faculty III, Hochschule Hannover University of Applied Sciences & Arts, Hannover, Lower Saxony Germany; 9 Clinical Trial Consortium Research Group, University of Nigeria, Enugu Campus, Enugu, Enugu State Nigeria; 10 Department of Physiotherapy, Faculty of Health Sciences, David Umahi federal University of Health Sciences, Uburu, Ebonyi State Nigeria; 11 UNIRED Research Group, Faculty III, Hochschule Hannover University of Applied Sciences & Arts, Lower Saxony 30159 Hannover, Germany

**Keywords:** Occupational-specific variations, Cardiorespiratory function, Haematological parameters, Cement workers, Woodworkers, Automobile spray painters, Cleaners

## Abstract

**Background:**

Upsurge in cardiopulmonary dysfunctions in Enugu, Nigeria, involved mainly cement workers, automobile spray painters, woodworkers, and Cleaners and was worsened in the dry season, suggesting the need for an occupation-specific characterization of the disease features and seasonal evaluation of air quality for prevention and management.

**Methods:**

We conducted a randomized cross-sectional study of eighty consenting participants (in Achara Layout, Enugu), comprising 20 cement workers (39.50 ± 14.95 years), 20 automobile spray painters (40.75 ± 9.85 years), 20 woodworkers (52.20 ± 9.77 years), and 20 cleaners (42.30 ± 9.06 years). The air quality, some haematological (fibrinogen-Fc, and C-reactive protein-CRP), and cardiopulmonary parameters were measured and analyzed using ANCOVA, at *p* < 0.05.

**Results:**

The dry season particulate matter (PM) in ambient air exceeded the WHO standards in the New layout [PM_10_ = 541.17 ± 258.72 µg/m^3^; PM_2.5_ = 72.92 ± 25.81 µg/m^3^] and the University campus [PM_10_ = 244 ± 74.79 µg/m^3^; PM_2.5_ = 30.33 ± 16.10 µg/m^3^], but the former was twice higher. The PM differed significantly (*p* < 0.05) across the sites. Forced expiratory volume at the first second (FEV_1_) (*F* = 6.128; *p* = 0.001), and Peak expiratory flow rate (PEFR) (*F* = 5.523; *p* = 0.002), differed significantly across the groups. FEV_1_/FVC% was < 70% in cement workers (55.33%) and woodworkers (61.79%), unlike, automobile spray painters (72.22%) and cleaners (70.66%). FEV_1_ and work duration were significantly and negatively related in cement workers (*r* = -0.46; *r2* = 0.2116; *p* = 0.041 one-tailed). CRP (normal range ≤ 3.0 mg/L) and Fc (normal range—1.5–3.0 g/L) varied in cement workers (3.32 ± 0.93 mg/L versus 3.01 ± 0.85 g/L), automobile spray painters (2.90 ± 1.19 mg/L versus 2.54 ± 0.99 mg/L), woodworkers (2.79 ± 1.10 mg/L versus 2.37 ± 0.92 g/L) and cleaners (3.06 ± 0.82 mg/L versus 2.54 ± 0.70 g/L).

**Conclusion(s):**

Poor air quality was evident at the study sites, especially in the dry season. Cement workers and automobile spray painters showed significant risks of obstructive pulmonary diseases while woodworkers had restrictive lung diseases. Cement workers and cleaners recorded the highest risk of coronary heart disease (CRP ≥ 3.0 mg/L). The similarity in Fc and CRP trends suggests a role for the inflammation-sensitive proteins in the determination of cardiovascular risk in cement workers and cleaners. Therefore, there are occupation-specific disease endpoints of public health concern that likewise warrant specific preventive and management approaches among the workers.

## Background

Air pollution is a very important occupational problem in various workplaces and poses a threat to health. Air pollution occurs when any substance is found in the ambient air at concentrations that far exceed the normal levels and may be injurious to property, plants, animals and humans [1–3]. Air pollutants are derived from a heterogeneous complex mixture of gases, liquids and particulate matter, [4, 5] and increase with industrialization [6]. Exposures to dust, fumes, and gases such as Portland cement dust, wood dust, and isocyanate paint mist are associated with an increased prevalence of respiratory symptoms and ventilatory impairments [7–11]. Significant decreases in forced expiratory volume (FEV_1_), forced vital capacity (FVC) and peak expiratory flow rate (PEFR), are associated with exposures to gases and fumes resulting in respiratory symptoms including cough, dyspnea and phlegm [12,13]. Although the lungs may not be adversely affected by some pollutants, however, it provides the means through which the pollutants enter the bloodstream and harm other organs or impair the blood’s oxygen-carrying capacity [12]. The aforementioned studies suggest that air pollutants are occupation-specific, and likewise the disease patterns. Therefore, the preventive and management interventions should be occupation-specific.

**Fig. 1:**
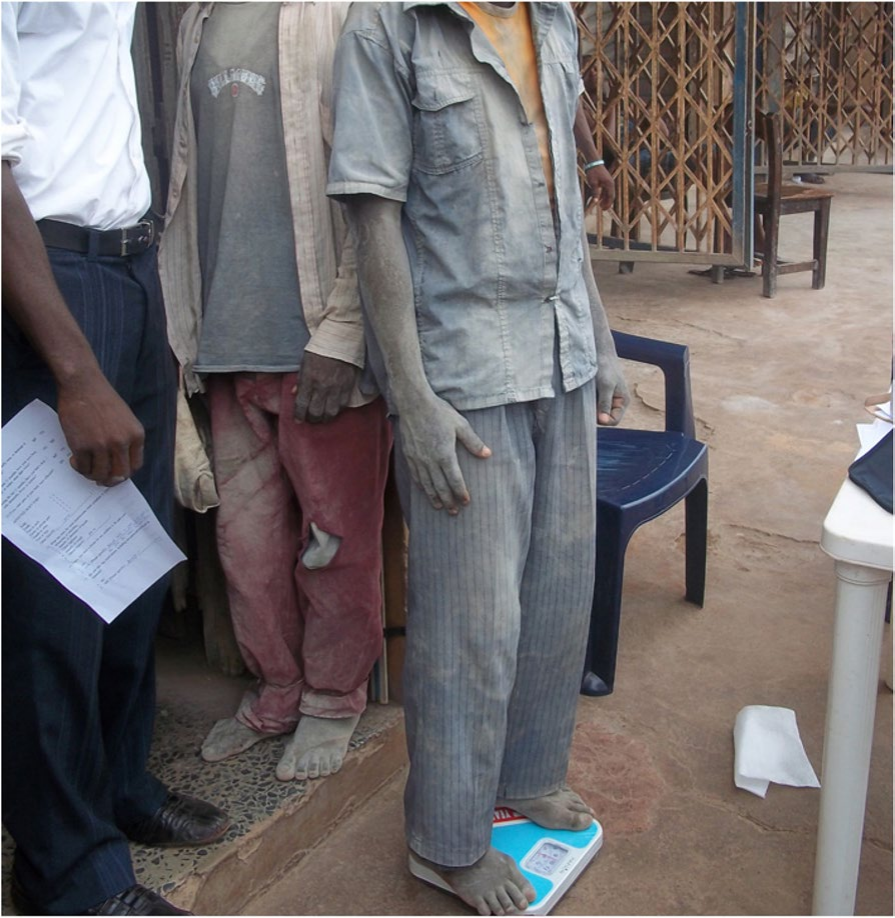
Cement workers covered in cement dust without protective gears to prevent inhalation and body contact with pollutants at the workplace

As most developing countries build modest economic growth and aim towards increased urbanization, infrastructural development and industrialization, [14, 15] little attention is given to the negative impact on environmental quality and associated health issues. Enugu metropolis, in South-East Nigeria, falls into the category of rapidly developing urban cities, and has been selected as one of the 5 resilient cities in Africa by the Rockefeller foundation [16]. Such tempo of development implies an increase in the number of residents exposed to airborne pollution from industrial activities. This could explain the upsurge in cases of cardiopulmonary diseases at the out-patient unit of the Physiotherapy Department, University of Nigeria Teaching Hospital (UNTH), Ituku-Ozalla, Enugu. Most of the patients were cement workers, automobile spray painters, woodworkers and Cleaners. For instance, 13 patients were treated with various cardiopulmonary conditions in 2007 whereas, in 2011, 34 patients were treated, which represented over 250% increase in the prevalence rate. The public health team was therefore prompted to visit their workplaces and observed that some of them were directly exposed to paint mist/fumes, wood dust and cement dust without protective gear, and adherence to caution (Fig. 1). It was observed that workers carried cement bags on their heads without any protective measures or mechanized equipment to prevent inhalation and bodily contact [17]. This is worrisome because cement workers in Europe reported adverse health conditions despite strict adherence to protective cautions [18, 19].

In populations exposed to air pollution, blood viscosity, fibrinogen (Fc), and C-reactive protein (CRP) were higher, and are forerunners of fatal cardiovascular events [20]. Air pollution is associated with increased heart rate [21, 22] and is most marked in individuals who had high blood viscosity [22]. Some airborne chemicals stimulate the immune system to activate leukocytes and macrophages leading to tissue damage especially in the cells that line human blood vessels [23, 24]. Sustaining the above cardiovascular and haematological changes could lead to hypertension and eventually ischaemic heart disease [25]. Overall, the above scenarios highlight the need to understand the relationships between air quality and diseases among workers because air pollution is associated with high numbers of respiratory and cardiovascular hospital admissions, bronchitis episodes, and restricted activity days [26–28]. which should reduce workers' productivity with negative economic consequences in developing economies like Nigeria. Therefore, we sought to i. Determine the differences in air quality (particulate matter concentration) at workplaces in the market (for cement workers, Automobile spray painters, and woodworkers) compared to university (non-market area for cleaners) where workers with respiratory conditions were identified, ii. Determine the variation in lung function parameters among the workers (cement workers, Automobile spray painters, woodworkers and cleaners), iii. Determine the variation in the haematological (C-reactive protein – CRP, and Fibrinogen – Fc) parameters among the workers, iv. Determine the relationship between work duration and lung function among the workers, and v. Determine the relationship between the lung function, and haematological parameters among the workers in the market area (cement workers, Automobile spray painters, woodworkers).

## Methods

### Population and study design

**Fig. 2:**
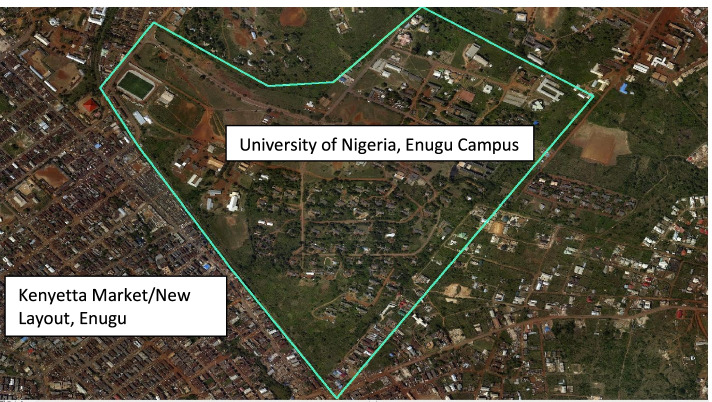
Aerial photograph showing the University of Enugu Campus and Kenyatta market lying adjacent. (Used with permission of the copyright owner—Okwuchukwu CN, Somtoochukwu CO, Amarachukwu O, Ndukwe EC, Chisom OE. Department of Geoinformatics and Surveying, Faculty of Environmental Studies, University of Nigeria Enugu Campus, Enugu, 4,000,001, Enugu State, Nigeria)

A randomized cross-sectional observational study was conducted, in New layout, Enugu, involving 80 consenting workers – comprising 20 cement workers, 20 automobile paint sprayers, 20 woodworkers and 20 cleaners, who were likewise selected from three faculties of the University of Nigeria, Enugu Campus, in New Layout, Enugu. New Layout Enugu has many industrial parks and was selected because most of our patients with cardiopulmonary conditions come from that area. Recruitment for the study was conducted at the Kenyatta market, which is known as the “building materials” market in the metropolis. With a 5% margin of error, at a confidence level of 95%, in a population of 123 workers, a response distribution of 50% was assumed, and a sample size of 94 was determined. Consenting workers were recruited immediately after a health talk given by the research team, at the weekly meeting of the trade union members, held at Kenyatta Market Fig. 2.

One major eligibility criterion was applied: 1. > 5 years of uninterrupted work experience at the same location. However, out of 123 workers in the market, only 75 workers with a self-reported history of cardiopulmonary conditions that met the eligibility criteria, were recruited, out of which 60 consenting participants were recruited by computer-generated simple random sampling method while providing for almost equal representation of the workers. They were recruited at the venue of their weekly meeting, using a membership list of all registered workers obtained from trade union leaders, who gave their prior permission. Similarly, 20 out of 69 cleaners (staff of the University of Nigeria, Enugu Campus), were recruited at their respective faculties using the same approach.

### Procedure

The study process involved four stages: obtaining informed consent, administering the questionnaire, cardiopulmonary assessment and laboratory study.

#### PM Measurement site selection and Ambient air monitoring protocol

The designated study sites were at the centre of each location; and were determined according to the observed level of human activities, traffic volume and population density. The coordinates of each site (Kenyatta/Timber Market / New Layout: 6° 24′ 54.2412'' N, 7° 30′ 15.2028'' E; and UNEC: 6° 25′ 38.0568'' N, 7° 30′ 31.1796'' E) were also measured using the Garmin global position system (Model Etrex H, Taiwan). The ambient air quality was assessed by determining the concentration of the suspended particulate matter at each site using a photometric-laser counter (Aerocet Model 531–9800 Rev. C, Metone Inc. U.S.A). It provided a digital readout on its LCD screen and is capable of generating particle data count for eight sizes of particles (including PM_2.5_, and PM_10_) as well as total suspended particulate (TSP). The instrument is configured to derive the mass concentration for the sampled particulates and has been used in previous studies in Enugu metropolis, and other cities in Nigeria and Italy [29, 30]. Using the same instrument allows for the comparison of data across similar studies. The instrument determined the air quality when the sampler is held within the breathing zone, which is about 2 m above the ground [31] with the parameter knob turned on until the data is generated and displayed on the LCD screen. This procedure lasted for two minutes at a time, one day in a week, 12 h (6.00 am—6.00 pm) per day, and at an interval of thirty minutes for 12 weeks (3 months). The measurements were done in the dry season, from December 2018 to February 2019, and repeated in the wet season, from June–August, 2019. The average of the mean hourly readings of each variable in twelve hours for each day and week was determined. The overall mean values in 12 weeks were subsequently computed.

#### Spirometric assessment

Before the assessment, the procedures were demonstrated to acquaint participants with the spirometric test. They were instructed to wear loose clothing, which would not limit thoracic expansion and abdominal mobility. The instrument used Morris/Polgar prediction [31–33] in its spirometric measurement, the assessment protocol was observed in an upright sitting position, in the following steps: i) breathe in and out three times in a relaxed position, ii) breathe in as much as possible (TLC level), iii) breathe out as fast and as much as possible (Forced expiration), and iv) breathe in as fast and as much as possible (Forced inspiration). Subsequently, the PEFR, FVC, and FEV1 were obtained and recorded. The highest value recorded in three trials, with less than 5% deviation from the other readings, was used. The forced expiratory volume in one second (FEV1), as a percentage of the forced vital capacity (FEV1/FVC%), was also recorded.

#### Laboratory studies

Venous blood (5 ml) was taken from a peripheral vein on the arm of each subject using a hypodermic needle fitted to a syringe. Thereafter, 4.5 ml of blood was immediately transferred into sterile Trisodium citrate anticoagulant bottles and 0.5 ml into a plain bottle. The samples were later subjected to a clot-based assay to determine the serum concentration of fibrinogen as already described elsewhere, [34] while the CRP was determined using an immunoradiometric assay (IRMA), as already described elsewhere [35, 36].

### Covariates

#### Sedatives and anti-hypertensives

Some anti-hypertensives and anti-anxiety drugs are known to induce bronchi constriction, especially beta-adrenergic blockers [37]. Some of the identified anti-anxiety and anti-hypertensive drugs used among the participants according to the Anatomical Therapeutic Chemical (ATC) classification system [38] included drugs acting on the nervous system (Group N) including N05A – Anti-psychotics, N05BA – Anxiolytics (benzodiazepine-derivatives), N05CD-Hypnotics, sedatives (benzodiazepine-derivatives) and anti-hypertensives (and their combinations) including—C03—Diuretics, C07—Beta blocking agents, C08—Calcium channel blockers, C02AB—methyldopa, and C09—Agents acting on the renin-angiotensin system.

#### Age

Some of the pulmonary function parameters measured in this study (such as FVC, FEV(1), FEV(1)/FVC, and PEFR), are known to decrease significantly with age in both males and females [39, 40].

#### Weight

Pulmonary function parameters (PEFR, FVC, FEV1, FEV1/FVC) vary significantly across various classes of body mass index [40, 41].

#### Work duration

Work duration has been associated with changes in pulmonary function in woodworkers [42], automobile spray painters [43], cement workers [44] and cleaners (sweepers) [45].

### Statistical analysis

Data were presented as means and standard deviations. All clinical and laboratory data were subjected to one-way ANCOVA, and differences between individual means were tested by a *Bonferroni* multiple-range test, after controlling for the extraneous variables, using the faculty Vassar computational website software programme. The Pearson correlation coefficient was used to determine the relationships between the variables. Tests of significance were performed using Faculty Vassarstats computation software (website), and alpha was set at *p*-value ≤ 0.05.

## Results

**Fig. 3:**
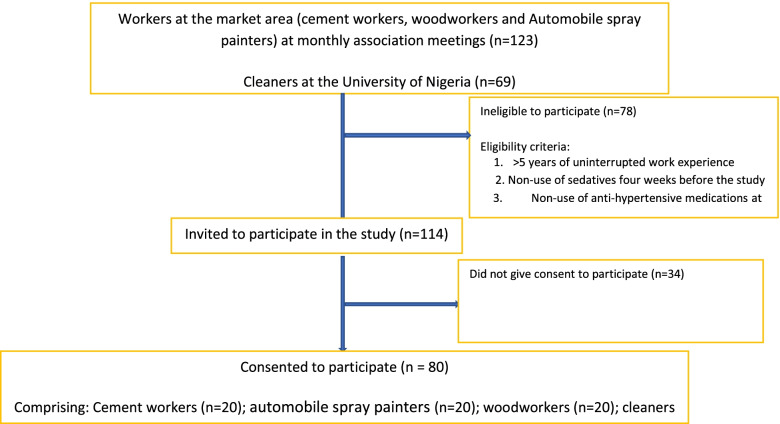
Design and flow of participants through the study

The participants’ flow through the study is presented in Fig. 3. Participants’ characteristics differed significantly across the groups (Table 1) for age (F(3,76) = 5.386; *p* = 0.002), weight (F(3,76) = 5.064; *p* = 0.003) and work duration (F(3,76) = 6.419; *p* = 0.001). The participants' socio-demographic characteristics were as follows:

i. *Cleaners:* The cleaners had a mean age of: 42.30 ± 9.06 years; age range: 23—53 years, mean height: 1.72 ± 0.06 m; height range: 1.56—1.82 m; mean weight: 79 0.60 ± 11. 38 kg; and weight range: 49 – 105 kg.
ii. *Cement workers:* The cement workers (Fig. 1) had a mean age of: 39.50 ± 14.95 years; age range: 17 – 65 years; mean height: 1.67 ± 0.79 m; height range: 1.51—1.83 m; mean weight: 67.65 ± 8.35 kg; and weight range: 53—85 kg.
iii. *Automobile Spray painters:* The automobile spray painters had a mean age of: 40.75 ± 9.85 years; age range: 28– 62 years; mean height: 1.73 ± 0.79 m; height range: 1.60—1.90 m; mean weight: 71.80 ± 11.50 kg; and weight range of 59 – 100 kg. The commonly used paint colours by automobile spray painters according to their local demand included ash, grey, black, and navy blue. (Table 2).
iv. *Woodworkers:* The mean age of: 52.20 ± 9.77 years; age range: 34 – 71 years; mean height: 1.69 ± 0.08 m; height range: 1.58 m -1.90 m; mean weight: 78.65 ± 13.48 kg; and weight range: 51–102 kg. The commonly processed woods and their uses by local woodworkers are listed in Table 2 according to priority in their local demand and include iroko/Orji (*Milicia excelsa*), Mahogany (*Khaya*) and Agba (*Prioria balsamifera*), and Masonia (*Mansonia altissimo*).

**Table 1:** Socio-demographic characteristics of the Participants (*n* = 80)

Variable	Category	Mean ± SD	Range	*F* – Value	*P* – value
Age (Years)				5.386	0.002*
	Civil servants	42.30 ± 9.06	23 – 53		
	Cement workers	39.50 ± 14.95	17 – 65		
	Spray Painters	40.75 ± 9.85	28 – 62		
	Woodworkers	52.20 ± 9.77	34 – 71		
Weight (Kg)				5.064	0.003*
	Civil servants	79.60 ± 11.38	49—105		
	Cement workers	67.65 ± 8.35	53—85		
	Spray Painters	71.80 ± 11.50	59—100		
	Woodworkers	78.65 ± 13.48	51 – 102		
Height (m)				2.250	0.089
	Civil servants	1.72 ± 0.06	1.56 – 1.82		
	Cement workers	1.67 ± 0.79	1.51 – 1.83		
	Spray Painters	1.73 ± 0.79	1.60 – 1.90		
	Woodworkers	1.69 ± 0.08	1.58 – 1.90		
Duration of Work (Years)				6.419	0.001*
	Civil servants	11.85 ± 8.96	4.00 – 31.00		
	Cement workers	10.15 ± 10.07	1.00 – 30.00		
	Spray Painters	19.10 ± 9.80	4.00 – 41.00		
	Woodworkers	22.25 ± 11.73	5.00 – 48.00		
**Demographic Information**				**Frequency**	**Percentage**
Academic Qualification					
	Not educated			03	05.00
	FSLSC			29	48.33
	SSCE			18	30.00
	National Vocational Certificate			05	08.33
	National Technical Certificate			02	03.33
	Advanced National Technical certificate			03	05.00
Category of skillset					
	Unskilled Labourers			33	55.00
	Skilled Labourers			10	16.67
	Foremen			09	15.00
	Vendors			07	11.67

*FSLC* First School Leaving certificate, *SSCE* Senior Secondary School Certificate, *spray painters* automobile spray painters

^*^Significant at *p* < 0.05

**Table 2:** Commonly used types of wood and paint colours by Woodworkers and automobile spray painters, respectively

Types of wood		Paints
**Common local (botanical0 Names**	**Uses**	**Commonly used paints**
Iroko/orji (*Milicia excelsa*)	Doors	Ash
Mahogany (*Khaya*)	Household/office furniture, cabinetry, boats, and mouldings, and used for all purposes because of their quality	Grey
Agba (*Prioria balsamifera*)	Bed floors and framework of chairs	Black
Masonia (*Mansonia altissima*)	Household/office furniture	Navy blue
*Gmelina Arborea*	Household/office furniture	Other shades of Blue
Akpu (*Ceiba Pentandra*)	Scaffolding in construction	Yellow
Ebenebe (*Sterculiaceae*)	Scaffolding in construction	Red
Ora (*Pterocarpus spp*)	Scaffolding in construction	White
Ube (*Dacryodes edulis*)	Door frames	Green
Western Red cedar *(Juniperus virginiana*)	Furniture	Purple
Pine (white, lodgepole, jack) *(Pinus contorta)*	lumber, plywood, and panelling	Brown
Obeche *(Triplochiton scleroxylon)*	veneer, furniture, picture frames and mouldings	
Teak	Furniture, flooring and interior fittings and decorations	

### PM concentration

#### Seasonal TSP concentration

Kenyatta/Timber Market's mean TSP concentrations were higher in the dry season than wet season (655.83 ± 395.29 µg/m3 versus 105.5 ± 37.93.05 µg/m3) similar to the UNEC study site (337.5 ± 157.90 µg/m3 versus 74.92 ± 22 µg/m3). (Table 3). However, in each season, the TSP mean concentration was significantly higher at the Kenyatta/Timber Market than UNEC (Dry season TSP mean difference = 318.33 µg/m3, t = 2.59; dF = 22; p = 0.008; Wet season TSP mean difference = 30.58 µg/m3; t = 2.41; dF = 22; *p* = 0.01). The TSP concentration in Kenyatta/Timber Market was twice as high as UNEC.

**Table 3:** Mean TSP, PM_10_ and PM_2.5_ concentrations at study sites

Sites	New layout/Kenyatta/Timbre Market						UNEC					
Coordinates	6° 24' 54.2412'' N						6° 25' 38.0568'' N					
	7° 30' 15.2028'' E						7° 30' 31.1796'' E					
WKs	TSP (µg/m^3^)		PM_10_ (µg/m^3^)		PM_2.5_ (µg/m^3^)		TSP (µg/m^3^)		PM_10_ (µg/m^3^)		PM_2.5_(µg/m^3^)	
	D	W	D	W	D	W	D	W	D	W	D	W
1	455	110	365	109	69	14	539	103	237	66	13	09
2	465	150	470	123	67	08	549	115	265	68	14	12
3	494	126	799	95	70	16	420	95	287	68	23	11
4	995	160	829	89	96	19	523	75	318	70	30	11
5	1540	108	878	79	129	09	450	89	319	64	59	09
6	1303	109	709	49	106	10	305	59	245	56	66	09
7	411	109	900	69	70	17	329	44	245	62	30	08
8	465	125	376	49	67	18	309	69	373	56	29	09
9	506	123	301	59	62	15	166	57	192	89	28	08
10	410	52	343	64	55	17	120	79	182	50	26	08
11	452	45	276	54	49	10	182	65	128	53	22	09
12	374	49	248	49	35	16	158	49	137	48	24	07
x̄	655.83	105.5	541.17	74.00	72.92	14.08	337.5	74.92	244	62.5	30.33	9.17
SD	395.29	37.93	258.72	25.12	25.81	3.82	157.90	22.05	74.79	11.18	16.10	1.47

*TSP* Total suspended particulate, *PM* Sizes of particulate matter, *UNEC* University of Nigeria, Enugu Campus, *D* Dry season, *W* Wet Season, *CRP* C - reactive protein, *x̄* mean, *SD* Standard deviation

#### *Seasonal PM*_*10*_* concentration*

Kenyatta/Timber Market recorded higher dry and wet season mean PM10 concentrations (541.17 ± 258.72 µg/m3 and 74.00 ± 125.12 µg/m3 (µg/m3) than UNEC (244 ± 74.79 µg/m3 and 62.5 ± 11.18 µg/m3), respectively (Table-3). Similarly, the mean difference in PM10 between the two sites was significantly higher in dry season (mean difF = 541.17 µg/m3, t = 3.82, dF = 22, *p* = 0.0005), unlike the wet season (mean difF = 11.5 µg/m3; t = 1.45; dF = 22; *p* = 0.08), respectively. The PM10 concentration at Kenyatta/Timber Market was twice as high as UNEC.

#### *Seasonal PM*_*2.5*_* concentration*

PM2.5 concentration varied seasonally similar to PM10 with relatively lesser values at UNEC, in both the dry (30.33 ± 16.10 µg/m3) and wet seasons (9.17 ± 1.47 µg/m3) compared to Kenyatta/Timber Market (72.92 ± 25.81 µg/m3 and 14.08 ± 3.82 µg/m3), respectively (Table 3). Similarly, the mean difference in PM10 concentration between the two sites was significantly higher in dry season (mean difF = 42.58 µg/m3; t = 4.85; dF = 22; *p* =  < 0.0001), than wet season (mean difF = 4.92 µg/m3; t = 4.16; dF = 22; *p* = 0.0002), respectively. The PM2.5 concentration at Kenyatta/Timber Market was twice as high as UNEC.

### Cardiopulmonary parameters

FEV_1_ (functional index of airway resistance) differed significantly (*F* = 6.13; *p* = 0.001) across the groups, and variations from the reference value in FEV_1_ occurred in 2.10% of the population (Table 4). Cement workers showed significantly lower adjusted mean FEV_1_ values (mean difference = 1.10L; SE = 0.29; *p* = 0.001; CI = -1.85 to -0.34) than the cleaners, and automobile spray painters (mean difference = -0.90L; SE = 0.28; *p* = 0.010; CI = -1.65 to—0.15), respectively. However, FEV_1_ was significantly higher in cleaners compared to woodworkers (mean difference = 0.90L; SE = 0.30; *p* = 0.021; CI = -1.71 to -0.09). The effect size among the woodworkers was moderate (d = 0.50), small for automobile spray painters (d = 0.20), and large for the cement workers (d = 1.10) compared to the cleaners. The post hoc analysis (Table 5), showed that the group differences in the FEV_1_ were accounted for by cement workers versus cleaners (*p* = 0.001; CI = -1.85 to -0.34), cement workers versus automobile spray painters (*p* = 0.01; CI = -1.65 to -0.15) and cement workers versus woodworkers (*p* = 0.02; CI = -1.71 to -0.92). The most superior effect on FEV_1_ was observed when comparing cement workers to cleaners. Adjusted mean FEV_1_ was lower in cement workers than cleaners and automobile spray painters, respectively unlike woodworkers.

**Table 4:** Variation in lung function, cardiorespiratory, and haematological Parameters of the Participants (*n* = 80)

Variables	Cleaners (*n* = 20)	Cement workers(*n* = 20)	Spray Painters(*n* = 20)	Woodworkers (*n* = 20)	*F*-value	*p-value*
**Lung Function Parameters**						
FVC(L)	3.34 ± 3.45	2.44 ± 1.86	3.06 ± 0.72	3.01 ± 2.47	0.10	0.40
FEV_1_(L)	2.36 ± 1.13	1.35 ± 0.72	2.21 ± 0.87	1.86 ± 0.98	6.13	0.001**
PEFR(L/min)	3.74 ± 1.71	2.49 ± 1.47	4.40 ± 1.71	3.12 ± 1.31	5.52	0.002**
FEV_1_/FVC(%)	0.71 ± 5.6	0.55 ± 14.2	0.72 ± 8.0	0.62 ± 11.0	0.85	0.10
**Haematological Parameters**						
Fc(mg/dL)	2.54 ± 0.70	3.01 ± 0.85	2.54 ± 0.99	2.37 ± 0.92	2.67	0.054
CRP Level(mg/L)	3.06 ± 0.82	3.32 ± 0.93	2.90 ± 1.19	2.79 ± 1.10	0.86	0.46

*SBP* Systolic Blood Pressure, *DBP* Diastolic Blood Pressure, *X* mean, *SD* Standard deviation

^*^Significant at *p* < 0.05

*Keys*: *FVC* Forced Vital Capacity, *FEV*_*1*_ Forced Expiratory Volume in one second, *PEF* Peak Expiratory Flow rate

^*^Significant at *p* < 0.05

*Fc* Fibrinogen concentration, *CRP* C—reactive protein, *x̄* mean, *SD* Standard deviation

^*^Indicate significant at *p* < 0.05

^**^indicates significant at *p* < 0.001; spray painters = automobile spray painters

**Table 5:** *Post-hoc* Test for Forced Expiratory Volume in 1 s

Group	Mean Difference	Standard Error	*P-value*	95% CI
Cleaners Vs Cement workers	1.28*	0.28	0.001**	-1.85 to -0.34
Cleaners Vs spray Painters	0.42	0.28	1.00	-0.56 to 0.95
Cleaners Vs Woodworkers	0.77	0.29	1.00	-0.60 to 0.98
Cement workers Vs Spray Painters	-0.86*	0.28	0.01*	-1.65 to – 0.15
Cement workers Vs Woodworkers	-0.51*	0.30	0.02*	-1.71 to -0.09
Spray Painters Vs Woodworkers	0.35	0.30	1.00	-0.80 to 0.80

*Keys*: *Vs* Versus, *CI* confidence interval, *spray painters* automobile spray painters

^*^The mean difference is significant at the 0.05 level

The PEFR (measure of airway caliber) differed significantly (Table 6) across the groups (F(3, 75) = 5.52; *p* = 0.002), with cement workers (mean difference = 1.34L; SE = 0.49; *p* = 0.049; CI = 0.01 – 2.68) and automobile spray painters (mean difference = -1.95; SE = 0.42; *p* = 0.001; CI = -3.28 to -0.62) having a significantly lower adjusted mean PEF rate than cleaners. PEFR varied in 17.10% of the sample compared to the reference value with a moderate effect size in woodworkers (d = 0.40) and automobile spray painters (d = 0.40), with a large effect size in cement workers (d = 0.80). The post hoc analysis (Table 6), showed that the group differences in the PEFR were accounted for by cement workers versus cleaners (*p* = 0.049; CI = -2.68 to -0.01), and cement workers compared to automobile spray painters (*p* = 0.001; CI = -3.28 to -0.62 The most superior effect on PEFR was observed when comparing cement workers to automobile spray painters. Adjusted mean FEV_1_ was lower in cement workers than in cleaners and automobile spray painters, respectively.

**Table 6:** *Post-hoc* Test for Peak Expiratory Flow

Group	Mean Difference	Standard Error	*P-value*	95% CI
Cleaners Vs Cement workers	1.25*	0.49	0.049*	0.01 to 2.68
Cleaners Vs Spray Painters	-0.66	0.49	1.00	-1.94 to 0.72
Cleaners Vs Woodworkers	0.62	0.52	1.00	-1.08 to 1.72
Cement Vs Spray Painters	-1.91*	-0.42	0.001*	-3.28 to -0.62
Cement Vs Woodworkers	-0.62	0.53	0.35	-2.46 to 0.42
Spray Painters Vs Woodworkers	1.28	0.52	0.48	-0.49 to 2.35

*Keys*: *Vs* Versus,  *CI* confidence interval, *spray painters *automobile spray painters

^*^The mean difference is significant at the 0.05 level

FVC (measure of lung volume) did not differ significantly (*F* = 0.995; *p* = 0.40) across the groups, and the effect size was small in cement workers (d = 0.30), automobile spray painters (d = 0.10) and woodworkers (d = 0. 11), respectively. However, compared to the reference value, the variation in FVC across the groups occurred in 2.80% of the sample. In addition, FEV1/FVC% values across the groups were as follows: cleaners = 70.66%; Cement workers = 55.33%; automobile spray Painters = 72.22%; and woodworkers = 61.79%. Further analysis of the cardiorespiratory profile within each group showed that 55%, 50%, 40% and 25% of the cement workers, woodworkers, cleaners and automobile spray painters had FEV1/FVC values < 70%, respectively < 70%, which is typical of obstructive lung diseases.

### The serum concentration of biomarkers of inflammation and coagulability

The adjusted mean CRP serum concentration showed no significant variation across the groups (*F* = 0.86; *p* = 0.46) (Table 4). However, the mean serum concentration of CRP was > 3.00 mg/L among cement workers (3.32 mg/L) and cleaners (3.02 mg/L), but > 2.0 mg/L and < 3.0 mg/L in automobile spray painters (2.90 mg/L) and woodworkers (2.79 mg/L). CRP level varied only in 1.3% of the workers when compared to the cleaners. There was small effect size for CRP variation in cement workers (d = 0.30), automobile spray painters (d = 0.20) and woodworkers (d = 0.30) compared to cleaners. The blood concentration of the measures of plasma viscosity, and possibly blood coagulability (Fc), did not vary significantly across the groups (*F* = 2.67; *p* = 0.054) and the respective effect size was small in woodworkers (d = 0.20), and paint mist (d = 0.001) unlike moderate effect recorded in cement workers (d = 0.60). The Fc value was highest in cement workers similar to the cleaners and paint workers, respectively, and the least being the woodworkers.

### Relationships between work duration vs lung function

Cement workers had a significant negative correlation (Table 7) between FEV_1_ and work duration (*r* = -0.46; *r2* = 0.21; *N* = 20; *p* = 0.04, one-tailed), unlike automobile spray painters, cleaners and woodworkers (*p* > 0.05).

**Table 7:** Relationship between work duration, and lung function (*n* = 80)

Variables	Cement workers (*n* = 20)	Spray Painters (*n* = 20)	Woodworkers (*n* = 20)	Cleaners (*n* = 20)
	*r-*value(*p-*value)	*r-*value(*p-*value)	*r-*value(*p-*value)	*r-*value(*r-*value)
**Duration of Work and Lung Function Parameters**				
FVC(L)	-0.36 (0.13)	0.33 (0.16)	0.16(0.51)	-0.26(0.26)
FEV_1_(L)	-0.46 (0.04*)	-0.15 (0.53)	0.14 (0.57)	-0.38 (0.10)
PEFR(L/min)	-0.42 (0.07)	-0.01 (0.97)	0.35 (0.14)	-0.34 (0.14)
**Duration of Work and serum level of inflammatory proteins**				
Fc(mg/dL)	-0.35 (0.13)	0.28 (0.24)	0.21 (0.39)	0.10 (0.58)
CRP Level(mg/L)	-0.35 (0.13)	0.19 (0.42)	0.07 (0.78)	0.10(0.69)

*Keys*: *FVC* Forced Vital Capacity, *FEV1* Forced Expiratory Volume in one second, *PEF* Peak Expiratory Flow rate

^*^Correlation is significant at the 0.05 level; *Fc* Fibrinogen concentration, *CRP* C-reactive Protein, *Spray painters* automobile spray painters

The PEFR and FVC were not significantly (*p* > 0.05) related to the work duration across the groups, respectively. The CRP and fibrinogen levels were not significantly related (*p* > 0.05) to the work duration of all the workers (Table 8).

**Table 8:** Relationship between lung function, and haematological Parameters in woodworkers, paint workers and cement workers (*n* = 60)

Variables	Cement workers (*n* = 20)		Spray Painters (*n* = 20)		Woodworkers (*n* = 20)	
	*r-*value(*p-*value)		*r-*value(*p-*value)		*r-*value(*p-*value)	
**Lung Function & haematological parameters**	Fc	CRP	Fc	CRP	Fc	RP
FVC (L)	.03(.91)	.02(.92)	-.16(.50)	-.17(.49)	-.16(.51)	-.24(.31)
FEV_1_(L)	.18(.46)	.18(.45)	-.28(.24)	-.24(.31)	-.16(.50)	-.17(.48)
PEFR(L/min)	.22(.35)	.22(.34)	-.26(.22)	-.30(.20)	-.23(.34)	-.25(.29)

*Keys*: *FVC* Forced Vital Capacity, *FEV1* Forced Expiratory Volume in one second, *PEF* Peak Expiratory Flow rate, *Fc* Fibrinogen concentration, *CRP* C—reactive protein

^*^indicates that correlation is significant at *p* < 0.05

^**^indicates that correlation is significant at *p* < 0.001 level; *spray painters* automobile spray painters

## Discussion

The seasonal mean PM_10_ levels (62.5 ± 11.18 µg/m^3^ to 541.17 ± 258.72 µg/m^3^) at both study sites (Kenyatta/Timber Market and UNEC) exceeded the WHO annual average guideline value of 20 µg m^−3^ and was above the values reported in some cities within and outside Nigeria [46, 47]. In contrast, the recorded PM_2.5_ concentrations were within the time-weighted permissible limit of 10–340 µg/m^3^ reported by other authors [48] who observed three sites in urban Guatemala during the summer season. Comparatively, such cities are smaller than Enugu in terms of size, traffic volume, population density and built-up areas, and may explain the activities that often lead to the higher concentration of PM in the environment. Technically, any amount of particulate matter in the air is not safe, however, the United States Environmental Protection Agency (EPA) [49] recommends that the best way to prevent any short or long-term health effects from developing is to limit exposure to PM_2.5_ concentrations below a short-term standard (24-h or daily average) of 35 µg per cubic metre of air (µg/m3) or a long-term (annual) standard of 12 µg/m^3^. Since our study lasted for a 3-month duration, it is reasonable to use the EPA long-term standard in interpreting the findings than short-term standards. Consequently, the wet season’s mean concentration of PM_2.5_ at the UNEC study site was within (9.17 ± 1.47 µg/m^3^) of the United States Environmental Protection Agency [49] recommended long-term standard of 12 µg/m^3^ unlike the Kenyetta market study site (14.08 ± 3.82 µg/m^3^). However, the dry season means PM_2.5_ concentration at both sites (Kenyetta market—72.92 ± 25.81 µg/m^3^, and UNEC -30.33 ± 16.10 µg/m^3^) exceeded the long-term permissible limits.

Based on the interpretation of PM_2.5_ values relative to health outcomes as indicated in the updated 2012 EPA standards [50], the PM_2.5_ value of 72.92 µg/m^3^ recorded in Kenyatta/Timber Market was unhealthy (55.5 – 150.4 µg/m^3^), while the value (16.10 µg/m^3^) recorded at UNEC was moderate (12.1 – 35.4 µg/m^3^), but not unhealthy for sensitive groups (35.5 – 55.4 µg/m^3^). This implies that the dry season could be associated with the aggravation of public health issues related to poor air quality, in both the Kenyatta/Timber Market area and UNEC. The health implications of these findings were varied and related to the size of the PM. The coarse particles (also technically defined as PM_10-2.5_) are inhaled into the upper respiratory tract, while the finer PM_2.5_ are inhaled deeply into the lungs (alveoli), [51] resulting in the incidence and severity of various respiratory diseases. This implies that the elevated PM_2.5_ levels in the dry season should be of public health concern. The study sites in Enugu when compared to some other cities in Nigeria, may attract greater public health concerns since the PM was higher than the annual mean of 123.6 µg/m^3^ recorded in some cities in Nigeria [30, 47, 52].

The cement workers’ plasma CRP—a biomarker of systemic inflammation, [53] – was higher (CRP > 3.32 mg/L—indicating high-grade inflammatory state) than automobile spray painters (CRP > 2.9 mg/L – indicating moderate grade inflammatory state), and woodworkers (CR*P* = 2.79 mg/L – indicating moderate grade inflammatory state). Similarly, the cleaners (cleaners) experienced a high-grade inflammatory state (hence CRP > 3.06 mg/L). The CRP levels may also allude to other underlying adverse clinical events. For instance, the values of CRP > 1.0 mg/L but < 2.0 mg/L, represent a minimum risk for coronary heart disease (CHD), whereas values > 2.0 mg/L are indicative of moderate risk of CHD, but ≥ 3.0 mg/L represents the highest risk for CHD [54, 55]. Therefore woodworkers and automobile spray painters were at moderate risk of CHD, whereas cement workers and cleaners were at the highest risk of CHD, and should be monitored regularly by public health experts.

Woodworkers, automobile spray painters and civil servants’ serum Fc values were within the normal range (1.5–3.0 g/L) unlike cement workers (3.01 g/L). Therefore, cement workers showed a tendency toward higher plasma viscosity and hence greater risk of myocardial infarction [56, 57]. Overall, the trend in Fc is similar to CRP and could suggest that these inflammation-sensitive proteins might have a role in the determination of cardiovascular risk, therefore can modify the strength of plasma level of Fc to predict the cardiac events associated with occupational exposure to pollutants.

Lung function was not significantly related to either CRP or Fc and which does not imply a lack of effect but rather that there could be some other non-vascular trigger for intrapulmonary inflammation that may influence lung function. Also, the serum levels of CRP and Fc were not significantly related to work duration across the groups. This might suggest that the severity of lung dysfunction associated with work duration or occupational exposure to pollutants may be determined by other factors like the quantity and nature of the inhaled pollutants [58], rather than the duration of exposure to the pollutants. This agrees with our findings that there is a high concentration of PM at both study sites, which varied seasonally. All these findings strengthen our initial view that the increasing trends of respiratory disorders/diseases recorded among workers in the Enugu metropolis may be related to unhealthy exposure to airborne pollutants and might be occupation-specific.

The FVC was not significantly changed across the groups and might have diagnostic implications since FVC is a measure of restrictive, but not obstructive lung diseases [59]. The small effect size recorded when comparing the groups indicates that the variation in FVC is not of clinical relevance.

The lower FEV_1_ observed in cement workers and woodworkers compared to cleaners, respectively, could be evidence of small airway diseases, which should increase the functional airways resistance [59]. This is typical of obstructive airways diseases, [59] and suggests the possibility of allergic responses to inhaled pollutants, which appears more pronounced among cement workers and woodworkers. This is reasonable considering the lower values of FEV_1_ recorded among either cement workers or woodworkers and agrees with the findings of another study [60] that exposure to wood dust results in restrictive disease (20% and 6.8%), obstructive disease (17.10% and 5.70%) and mixed pattern (7.10% and 0%] among woodworkers in Ethiopia and control group respectively. The moderate effect size in woodworkers indicates that the findings are of clinical concern, but the large effect size in cement workers is of clinical significance.

Despite having a lower FEV_1_, the FEV_1_/FVC was > 70% in woodworkers in our study. This is a typical feature of restrictive lung disease [47]. In contrast, despite significant decreases in FEV_1_, the FEV_1_/FVC ratio was < 70% in cement dust and paint mist, which is typical of obstructive lung diseases, and was recorded in half of the cement workers (55%) and spray automobile spray painters (50%). The significant negative relationship between FEV_1_ and work duration in cement workers and cleaners (unlike in woodworkers and automobile spray painters) suggests the possibility of a progressive increase in airway obstruction and peripheral resistance to airflow, over the years.

Cement workers showed differences in lung function which also suggest the involvement of the central airways, such as the larynx, since PEFR was significantly lower among them than the automobile spray painters, woodworkers, and civil servants, respectively. PEFR measures the patency of central airways and under-estimates airflow limitations in the peripheral airways/obstructive diseases [59, 61]. Therefore significant obstructions of small and medium-sized pulmonary airways would occur before PEFR decreases. In airway diseases that progress from the smaller distal to larger proximal parts of the respiratory system, a decrease in PEFR is a late sign of airway obstruction. In essence, PEFR can be normal despite small and medium airway obstruction [62]. Consequently, decreased PEFR observed by cement workers could indicate a severe airway obstruction that has progressed beyond the small and medium airways, unlike what was obtained with automobile spray painters and woodworkers. The moderate effect size in woodworker and automobile spray painters, respectively, indicates that the findings are of clinical concern, but the large effect size in cement workers is of clinical significance.

The above trend is significant considering that the work duration of automobile spray painters and woodworkers was greater compared to cement workers, respectively. It suggests that exposure to cement dust was far more destructive than paint mist and wood dust; and might be a PM-related change, which is not dependent on the duration of exposure. This view is buttressed by the findings that there were no relationships between the work duration and PEFR, which should be of public health importance. In addition, since the PEFR is a measure of elastic recoil and pliancy of the lungs, it should decrease in fibrotic conditions and could involve hypertrophy of mucosal cells, probably due to irritation by dust. This might elicit greater secretion of mucous and formation of mucosal plugs likely to obstruct the exhaled air [59]. Therefore, differences in the lung function of cement workers observed in this study might involve fibrotic changes in the lung parenchyma tissues, unlike automobile spray painters and woodworkers. Previous toxicological and physiological projections, [63, 64] suggest that fine particles, like cement dust, may play a major part in determining human health for several reasons. First, they could be more noxious, since they include sulfates, nitrates, acids, metals, and particles with various chemicals adsorbed onto their surfaces. Second, compared to larger particles (e.g., wood dust), finer particles (e.g., cement dust – with PM_2.5_), can be inhaled more deeply into the lungs, remain suspended for a longer duration of time, infiltrate easily into indoor environments, and get transported over much longer distances [63].

### Strengths and weaknesses of the study

The primary strength of this study is its focus on assessing and validating the responses of various body systems, that are vulnerable to pollutants, with one another. Thus, the pulmonary differences found in workers with likely long-term occupational exposure to airborne pollutants were simultaneously investigated in a well-described population. In addition, advanced statistical modelling methods reinforced and related the findings to the variables of interest and eliminated the possibility of explaining the outcome using any other alternative hypothesis. The sample was profiled with well-characterized lung function parameters and biomarkers of systemic inflammation and coagulability of each participant.

This study also has some weaknesses, because it cannot establish cause and effect relationship. Given that this study was also designed to gain insight into responses of pulmonary function among workers who were likely to be occupationally exposed to pollutants, a longitudinal study design would have provided strong causal-effect evidence of work exposure to airborne pollutants and consequent changes in lung function. Such objective evidence can be established by tracking PEFR over duration at duty, and duration off-duty, or by a specific inhalation challenge test with specified extracts. Evidence of sensitization can likewise be established when a skin test returns positive or presence of specific IgE antibodies with the specified extracts. However, these were not done since the purpose of this study was not to establish a causal relationship, but to appraise the feasibility of a large-scale epidemiological study by observing time point variations in individuals unexposed and exposed to variables of interest that might improve our understanding of the nature of responses likely to occur in each context.

### Implications for care teams and policymakers

Various reliable pathological mechanisms have been proposed that give credence to the biological plausibility of the findings of our study. These include systemic oxidative stress [64], systemic inflammation [65], thrombosis [66] and coagulation [67], and vascular (including endothelial) dysfunction [68] – all of which have profound clinical relevance for healthcare providers. This understanding might have both diagnostic and prognostic value, which will guide the nature of intervention and rehabilitation in relevant cases. The greatest risk of coronary heart disease was among cement workers and civil servants. It suggests that beyond occupation-specific exposure to pollutants there is an environmental pollution concern that affects individuals who are not occupationally exposed to industrial pollutants in Enugu metropolis, and requires urgent public health action. Therefore, it brings to the fore the serious health implications of regulatory failure in ensuring that industries that produce airborne pollutants are not situated in residential and office districts, as currently obtains in New layout, Enugu Metropolis, in Enugu State, Nigeria, where this study was done. It was considered that chronic exposure to cement dust and paint mist, could worsen any established cardiopulmonary symptoms. Therefore, respiratory therapists and physicians must routinely examine and advise cement workers, automobile spray painters, woodworkers and Cleaners whose offices are located in a poorly regulated environment on the adverse consequences of prolonged occupational exposures to airborne pollutants. The need to live a healthy lifestyle at the population level must be advocated in government policies, matched with strict implementation of guidelines that aim to cut environmental pollution in residential areas and workplaces. Consequently, all the workers exposed to airborne pollutants should wear protective gear and exercise appropriate caution while at work to minimize bodily contact with pollutants. The findings of this study further highlight the need for respiratory therapists and physicians to periodically monitor all workers who are occupationally exposed to airborne pollutants for early detection and treatment of those at risk of respiratory conditions to forestall any health complications.

## Conclusion

There is evidence of poor air quality at the study sites, especially in the dry season. The endpoint of the health outcomes included significant dangers of ventilatory impairments, especially obstructive pulmonary diseases, in cement workers and automobile spray painters; and restrictive lung diseases in woodworkers. The trend in Fc is similar to CRP suggesting a role for the inflammation-sensitive proteins in the determination of cardiovascular risk but is of greater concern in cement workers and civil servants. Therefore, regulatory actions are required to control ambient air quality and minimize exposures to airborne pollutants. Public health promotion programmes must target workers at risk of serious clinical cardiovascular events that may arise from poor air quality at workplaces by improving the overall state of cardiovascular health in the population.

## Data Availability

The datasets generated and analyzed during the current study are not publicly available due to privacy and ethical concerns but are available from the corresponding author upon reasonable request.

## Abbreviations

PEFR: Peak expiratory flow rate
FVC: Forced vital capacity
FEV1: Forced expiratory volume at the first second
FEV1/FVC%: Forced expiratory volume in one second, as a percentage of the forced vital capacity
Fc: Fibrinogen
CRP: C-reactive protein
ECG: Electrocardiogram
UNTH: University of Nigeria Teaching Hospital
CHD: Coronary heart disease
PM: Particulate matter
MESA: Multi-Ethnic Study of Atherosclerosis
